# Clinical epidemiology and mortality risk factors of gastric cancer in a sub-Saharan African setting: a retrospective analysis of 120 cases in Yaoundé (Cameroon)

**DOI:** 10.11604/pamj.2020.37.104.25422

**Published:** 2020-09-30

**Authors:** Guy Aristide Bang, Eric Patrick Savom, Blondel Nana Oumarou, Cynthia Karelle Mboupda Ngamy, Georges Bwelle Moto, Yannick Mahamat Ekani Boukar, Pierre René Binyom, Arthur Essomba, Maurice Aurélien Sosso

**Affiliations:** 1 Department of Surgery and Subspecialties, Faculty of Medicine and Biomedical Sciences, University of Yaoundé I, Yaoundé, Cameroon,; 2 Surgical Unit, Yaoundé University Teaching Hospital, Yaoundé, Cameroon,; 3 Surgical Unit, Yaoundé General Hospital, Yaoundé, Cameroon,; 4 Digestive Surgery Unit, National Insurance Fund Health Centre, Yaoundé, Cameroon,; 5 Yaoundé Central Hospital, Yaoundé, Cameroon

**Keywords:** Gastric cancer, clinical epidemiology, survival, mortality risk factors, Cameroon

## Abstract

**Introduction:**

in sub-Saharan Africa, there is scare published data on cancer in general and gastric cancer in particular.

**Methods:**

we conducted a multicenter retrospective analysis of the medical records of patients followed for gastric cancer in 5 hospital departments in the city of Yaoundé (Cameroon) over 6 years.

**Results:**

we recorded a total of 120 patients with a mean age of 53.4 ± 13.7 years. There were 62 females (51.7%). The most common risk factors for gastric cancer in our patients was Helicobacter pylori infection (59 cases, 49.1%). Seventy-six patients (63.3%) consulted within 1 to 6 months of symptoms on set at the forefront of which chronic epigastralgia (74.1%). At endoscopy, the tumor was mostly located at the antrum and was locally advanced or metastatic in 25.8% and 58.4 of cases respectively. Adenocarcinoma was the main histologic type found in 105 (87.5%) cases. Curative treatment could only be implemented in 26.7% of patients. We noted a total of 85 deaths (70.8%) with a mean survival time of 5.91 ± 7.51 months. Survival rate at 3 and 5 years was 10.1% and 4.6%, respectively. On multivariable analysis, variables independently associated with overall survival included: WHO stage 3 performance status (p = 0.042), palpable epigastric mass on examination (p = 0.042), pyloric localization (p = 0.007), and liver metastasis (p = 0.012).

**Conclusion:**

clinical epidemiology of gastric cancer in our study is comparable to those of other African studies with a predominance of locally advanced/metastatic forms. Prognosis is grim with diagnostic delay behind all of the identified mortality risk factors.

## Introduction

Gastric cancer (GC) remains a major public health challenge worldwide. There is a significant geographic variation of its incidence [1]. Higher incidence rates are recorded in Western Asia (45.3/100000), Eastern Europe (24.6/100000), and South America (17.3/100000). Even though it moved from 4^th^ [2] to 6^th^ [1] rank of the most common cancer worldwide, GC remains the second leading cause of cancer deaths with 9.7 and 8.2% of the total cancer deaths in 2008 and 2018 respectively [1, 2]. The five-year survival rate of GC depends on the stage at diagnosis, ranging from 10% for advanced stages [3] to 90% for early diagnosis [4].

In Africa, attention is focused on communicable diseases and there is scarce published data on non-communicable diseases such as GC. The estimated incidence rate of GC in Africa is 4/100000 [2]. At the time of diagnosis, metastatic forms are predominant, ranging from 50 to 78.58% of the reported cases [5-7]. Indeed, there is a delay in consultation due to long and tortuous care pathways, especially out of the hospital, with traditional healers. The prognosis of GC in Africa is poor, with the 5-year survival rate ranging from 2.38 to 30% [7, 8].

To the best of our knowledge, very few studies in sub-Saharan Africa have studied the prognostic factors of GC and no study in our country has yet focused specifically on gastric tumors. We undertook this study to describe the clinical epidemiology and to determine the mortality risk factors of GC in Yaoundé, the capital of Cameroon, a sub-Saharan African setting.

## Methods

**Study design and setting:** we conducted a cross-sectional multicenter study in 5 hospital departments in the city of Yaoundé, the capital of Cameroon (central African sub-region): the General Surgery Unit of the Yaoundé University Teaching Hospital, the Gastroenterology Unit of the Yaoundé University Teaching Hospital, the Gastrointestinal Surgery Unit of the Central Hospital of Yaoundé, the Gastrointestinal Surgery Unit of the National Social Insurance Hospital, and the Medical Oncology Department of the Yaoundé General Hospital. In the city of Yaoundé, the three surgical units selected are the only university hospital departments dedicated to gastrointestinal surgery, and the medical oncology center selected is the only medical oncology center.

**Study participants:** we conducted a retrospective analysis of the medical records of patients followed for GC in the 5 selected departments, over 6 years, from January 2013 to December 2018. These patients were identified from consultation/hospitalization registers and operating reports. Their files were consulted to complete the standardized data collection form. The outcome of patients enrolled had to be known until January 2019. The diagnosis of GC had to be histologically confirmed. Duplicates, unusable files, tumors of the cardia, and files of patients lost to follow-up at the time of the study were excluded. Patients´ demographic, history, clinical, pathological, management, and outcome variables were collected.

**Sample size and statistical analysis:** a consecutive sample of all patients during the study period fulfilling inclusion criteria was considered for this study. Data analysis was conducted using IBM SPSS software for Windows, version 23.0 (IBM Corp, Armonk, New York, USA). Continuous variables with a normal distribution were described using means ± standard deviations (SD) while the medians and interquartile ranges (IQR) were used for skewed variables. Categorical variables were reported as counts and percentages. Kaplan-Meier survival analysis was used to build the survival curves of the study population. The sub-group comparison of sociodemographic and clinicopathologic variables was done using the Log Rank (Mantel-Cox) test. Univariate Cox proportional hazard models were used to identify potential predictor variables. Variables with a P-value< 0.01 in the univariate model were then included in a multivariate Cox proportional hazard regression model to identify independent predictors of survival and determine their respective hazard ratios (HR) with a 95% confidence interval. Factors with a P-value of less than 0.05 where considered independent predictors of survival.

## Results

**Figure 1:**
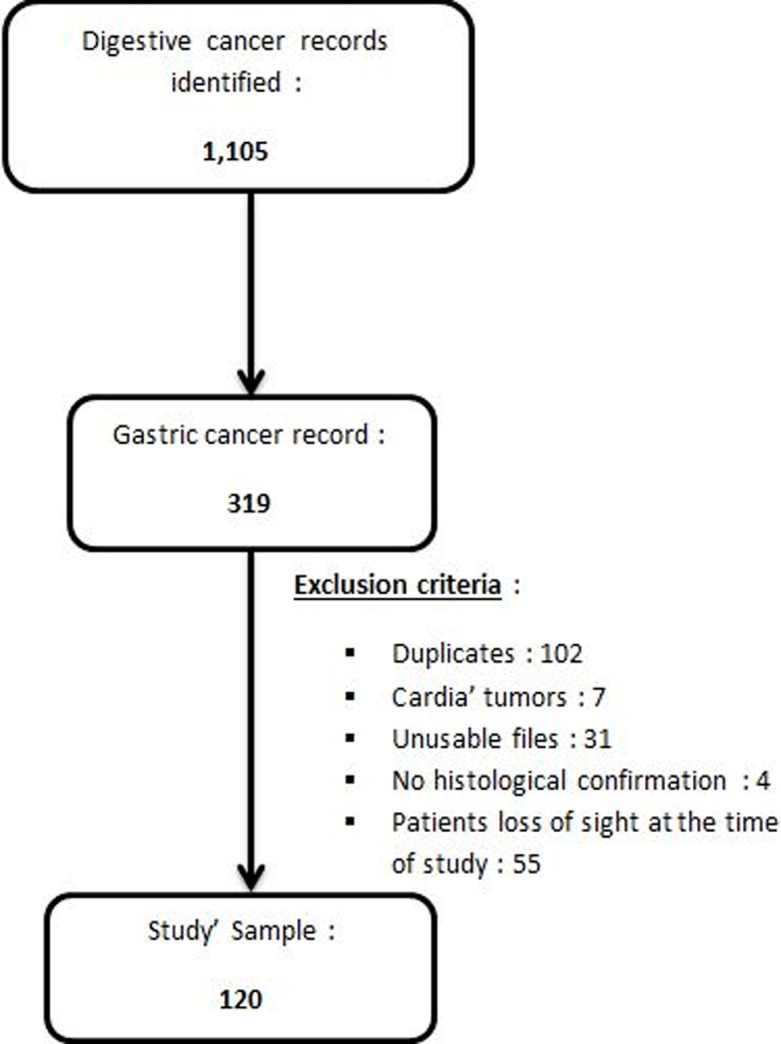
flowchart of patients´ selection

**Figure 2:**
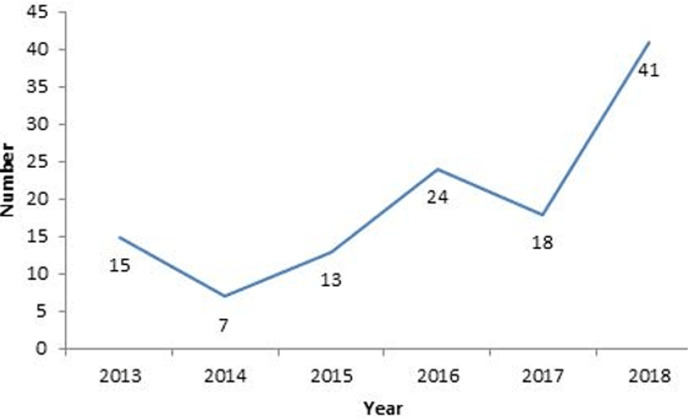
annual hospital incidence of gastric cancer cases

**Study participants:** a total of 1,105 patients with malignant digestive tumors were identified during the study period, among which 120 met our inclusion criteria. Figure 1 shows the flowchart of our sample selection. The annual incidence curve for GC cases (Figure 2) shows a general trend of gradual increase since 2014, with a peak in 2018 (41 cases).

**Figure 3:**
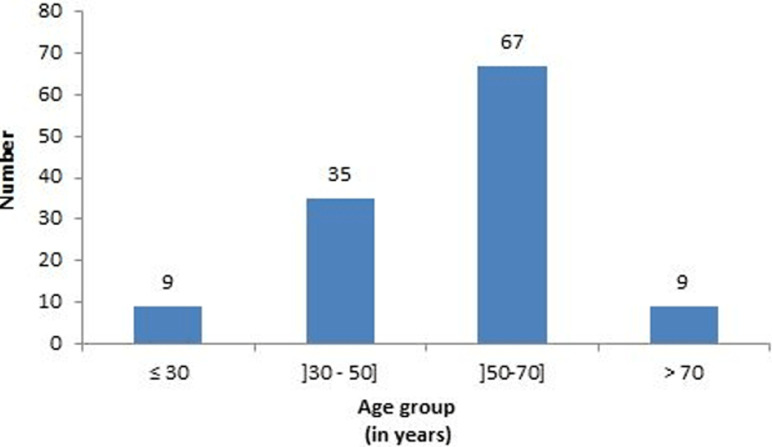
distribution of patients by age group

**Clinical epidemiology and therapeutic modalities:** there were 62 females (51.7%) and 58 males (48.3%) with a sex ratio of 0.94. Their mean age was 53.4 ± 13.7 years (range 21-89). The 50-70 years age group (Figure 3) was the most represented with 67 patients (55%). Table 1 resumes the clinical epidemiology and therapeutic modalities of our patients. The most common risk factors for GC in our patients were: *H pylori* infection (59 cases, 49.1%), chronic gastritis (49 cases, 40.8%), and consumption of smoked foods (37 cases, 30.8%). Seventy-six patients (63.3%) consulted within 1 to 6 months of symptom onset, while 38 (31.7%) did so in more than 6 months and only 6 (5%) in less than a month.In the vast majority of cases (86.7%), the discovery of GC was made after evocative symptoms at the forefront of which: chronic epigastralgia (74.1%), asthenia (73.3%), and weight loss (67.5%). However, 11.6% of cases were discovered following complications, including peritonitis by tumor perforation (0.8%). The general condition of patients was impaired in the majority of cases with 41.7% of them being in stage 3 of the WHO performance status and 38.3% in stage 4. Although weight loss was noted in 67.5% of patients, 20.1% of them were overweight or obese at the time of the diagnosis of GC. Anemia was the most common clinical sign (n = 56, 46.7%), a Troisier sign was noted in 25% of cases. The most common comorbidity was hypertension (11.7%). At endoscopy (n = 112, 93.3%), the tumor was mostly located at the antrum (n = 53, 44.2%) with a type III gross appearance according to Borrmann´s classification (n = 82, 68.3%). No endoluminal ultrasound was performed. The commonest imaging tool performed was abdominal CT-scan (n = 86, 71.7%). At the time of diagnosis, the GC was locally advanced or metastatic in 25.8% and 58.4 of cases respectively. Curative treatment could only be implemented in 26.7% of patients consisting of a subtotal gastrectomy. Adenocarcinoma was the main histologic type found in 105 (87.5%) cases.

**Table 1:** patients´ clinical epidemiology and therapeutic modalities

Variables	n (%)
**Signs on examination**	
Anemia	56(46.7)
Epigastric mass	43(35.8)
Ascites	30(25)
Troisier sign	30(25)
Jaundice	4(3.3)
**Comorbidities**	
Diabetes	8(6.6)
Hypertension	14(11.7)
HIV infection	8(6.7)
**The anatomic site of cancer at endoscopy (n=112)**	
Fundus	34(28.3)
Body	17(14.2)
Antrum	53(44.2)
Pylorus	2(1.6)
Overlap (diffuse form)	6(5.0)
**TNM classification**	
I	1(0.8)
II	14(11.6)
III	31(25.8)
IV	70(58.4)
Unspecified	4(3.4)
**Metastasis site**	
Liver metastasis	21(17.5)
Peritoneal metastasis	9(7.5)
Nodes involvement	36(30)
Pulmonary metastasis	2(1.6)
**Treatment**	
1- **Curative treatment**	32(26.7)
Curative surgery	32(26.7)
Neoadjuvant chemotherapy	10(8.3)
Adjuvant chemotherapy	30(25)
2- **Palliative treatment**	88(73.4)
Palliative surgery+chemotherapy	16(13.3)
Palliative chemotherapy	77(64.2)
**Histological appearance**	
Adenocarcinoma	105(87.5)
GIST	6(5)
Lymphoma	6(5)
Kaposi’s sarcoma	2(1.6)
Liposarcoma	1(0.8)
Leiomyosarcoma	1(0.8)

**Figure 4:**
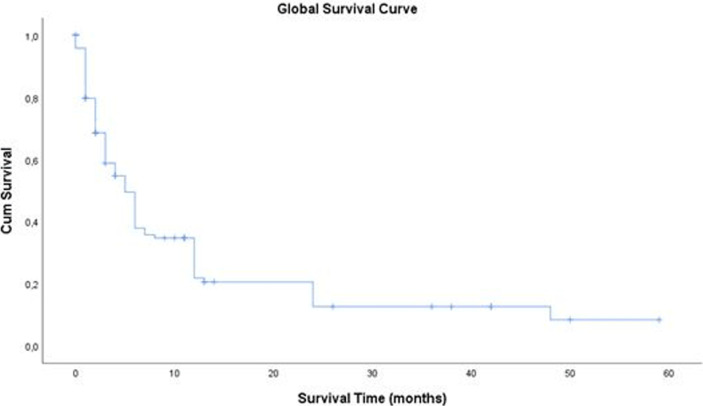
Kaplan-Meier curve of overall survival

**Figure 5:**
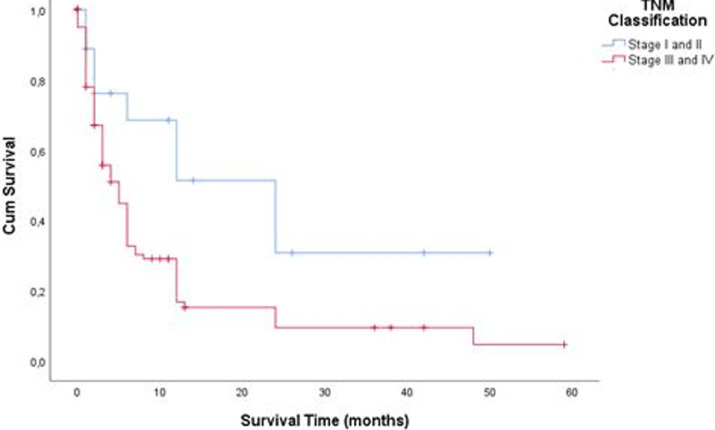
comparative Kaplan-Meier survival curve of early detected forms (TNM I and II) and others forms (TNM III and IV)

**Figure 6:**
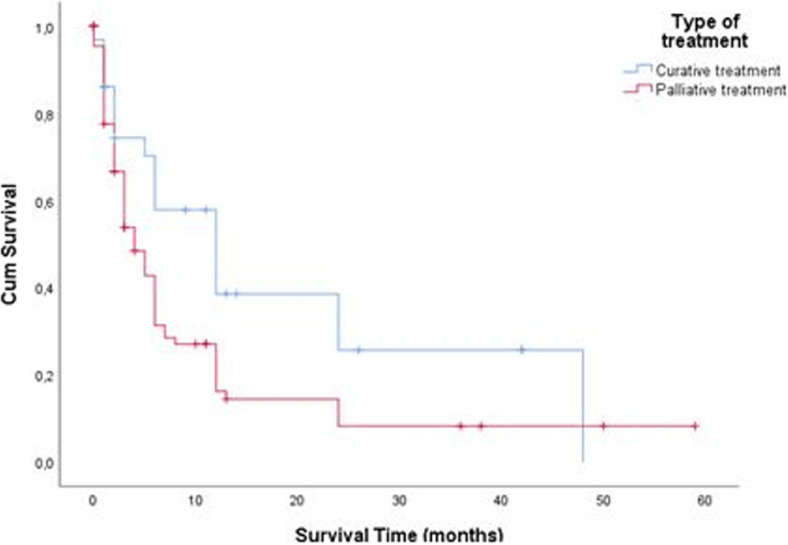
comparative Kaplan-Meier survival curve of patients with curative versus palliative treatment

**Survival:** we noted a total of 85 deaths (70.8%) with a mean survival time of 5.91 ± 7.51 months. The 5-year survival rate for the 22 patients operated in 2013 and 2014 was 4.6%. The 3-year survival rate for the 59 patients operated from 2013 to 2016 was 10.1%. Figure 4shows the Kaplan-Meier curve of overall survival (OS) of our patients. On univariate Cox proportional regression analysis (Table 2), 8 variables were associated with diminished overall survival: WHO stage 3 performance status (p = 0.007), palpable epigastric mass on examination (p=0.041),overlap (p=0.037) and pyloric localisation (p = 0.006), liver (p=0.001) and peritoneum metastasis (p = 0.021), TNM stage II (p=0.017), and curative treatment (p=0.032). On multivariable analysis (Table 3), variables independently associated with overall survival included: WHO stage 3 performance status (p = 0.042), palpable epigastric mass on examination (p = 0.042), pyloric localization (p = 0.007),and liver metastasis (p = 0.012). The survival of patients with early detected forms of GC (TNM I and II), was significantly better (log rank = 0.009) than that of other patients (TNM III and IV) as shown in Figure 5. Similarly, the survival of patients who received curative treatment was significantly better (log rank= 0.032) than that of those who received palliative treatment (Figure 6).

**Table 2:** univariate Cox proportional regression analysis to identify variables associated with overall survival

Variables	Deceased patient n(%)		Hazard ratio (IC 95%)		P-Value	
	Yes	No				
**Sex**						
Male	39(67.2)	19(32.8)	0.909(0.593-1.393)		0.661	
Female	46(74.2)	16 (25.8)	1		/	
**Delay between symptom onset and consultation**						
<1 month	4(66.7)	2(33.3)	0.665(0.207-2.134)		0.493	
1-6 months	52 (68.4)	24 (31.6)	0.605 (0.306-1.198)		0.149	
6-12 months	19(67.9)	9(32.1)	0.478 (0.220-1.038)		0.062	
**Comorbidities**						
Diabetes	3(50.0)	3(50.0)	1.454(0.456-4.640)		0.527	
Hypertension	8(57.1)	6 (42.9)	1.296 (0.624-2.693)		0.486	
HIV infection	5(62.5)	3(37.5)	0.706(0.285-1.747)		0.451	
**WHO performance status**						
1	1(50.0)	1(50.0)	0.344 (0.047-2.519)		0.294	
2	15(68.2)	7(31.8)	0.589(0.320-1.083)		0.089	
3	34 (68.0)	16 (32.0)	0.516(0.319-0.836)		**0.007**	
4	35(76.1)	11(23.9)	1		/	
**Obesity**	5(71.4)	2(28.6)	1.057(0.424-2.630)		0.906	
**Sign on physicalexamination**						
Palpable mass	34 (79.1)	9 (20.0)	0.631 (0.406-0.981)		**0.041**	
Troisiersign	22(73.3)	8(26.7)	1.603 (0.973-2.643)		0.064	
**Anatomic site**						
Body	10(58.8)	7(41.2)	2.819(0.742-10.697)		0.128	
Fundus	23 (67.6)	11 (32.4)	1.292 (0.574-2.904)		0.536	
Overlap (diffuse form)	5(83.3)	1(16.7)	3.236(1.070-9.783)		**0.037**	
Antral	42(79.2)	11 (20.8)	1.346(0.589-3.129)		0.555	
Pyloric	2(100)	0(0.0)	8.998(1.878-43.105)		**0.006**	
**Metastasis site**						
Nodeinvolvment	23 (63.9)	13 (36.1)	0.923 (0.540-1.578)		0.770	
Peritoneum	7 (77.8)	2 (22.2)	2.582 (1.150-5.794)		**0.021**	
Liver	20 (95.2)	1 (4.8)	2.530 (1.438-4.449)		**0.001**	
**TNM classification**						
Stade I	0 (0.0)	1 (100)	/		0.968	
Stade II	9 (50.0)	5 (90.0)	0.421 (0.207-0.859)		**0.017**	
Stade III	21 (67.7)	10 (32.3)	0.559 (0.308-1.017)		0.057	
IV	55 (78.6)	15 (21.4	1		/	
**Histologic type**						
Adenocarcinoma	76 (72.4)	29 (27.6)	1.852 (0.921-3.725)		0.084	
**Type of treatment**						
Curative surgery	18 (56.3)	14 (43.8)	0.562 (0.332-0.952)		**0.032**	
Palliative surgery	13 (81.3)	3 (18.8)	1.172 (0.648-2.118)		0.600	
Palliative chemotherapy	56 (72.7)	21 (27.3)	0.975 (0.621-1.532)		0.913	

**Table 3:** multivariable Cox proportional regression analysis to identify variables associated with overall survival

Variables	Hazard ratio (IC 95%)	P-value
WHO stage 3 performance status	1.935 (1.024-3.656)	0.042
Palpable mass	1.909(1.024-3.561)	0.042
Pyloric localization	0.020(0.001-0.352)	0.007
Overlap(diffuse form)	1.057(0.424-2.630)	0.906
Peritonealmetastasis	2.321(0.900-5.985)	0.081
Livermetastasis	2.310(1.206-4.422)	0.012
TNM stage II	1.301(0.481-3.519)	0.605
Curative treatment	0.751 (0.360-1.563)	0.443

## Discussion

Our study aimed to determine the clinical epidemiology and mortality risk factors of GC in a sub-Saharan African context. The limits of this study are linked to the retrospective nature of our data collection. Thus, of the 319 files of GC identified, only 120 met our inclusion criteria, with 55 files excluded because of patients´ unknown outcome. Despite these limits, we report 120 cases collected over 6 years. Other African studies report 36 cases collected in 7 years [8], 51 cases over 11 years [7], and 252 cases in 22 years [9]. All these data seem to confirm the low incidence of gastric cancer in Africa [2]. But in the absence of national cancer registries in most of these countries, lack of data collection, and under-reporting, this incidence rate may be underestimated. Gastric cancer in our series affects both sexes equally, with a slight predominance of women. Several African and Western studies have found a male predominance with a sex ratio sometimes reaching 5/1 [8, 10-12].

The mean age of our patients at the time of diagnosis was 53.4 years old, comparable to that reported in other African studies [7-9]. However, it should be high lighted that 44 patients (36.7%) were aged less than 50 years old. A hereditary component to gastric cancer in our environment can, therefore, be mentioned but the low availability of immuno-histochemical analyses and the absence of oncogenetic consultation do not allow us to support this hypothesis to date. *Helicobacter pylori* infection was the main risk factor for GC found in our patients. It´s not surprising to find a high prevalence of *Helicobacter pylori* infection in our context when we know that, one of the contributing factors of this infection is a low socio-economic level [13, 14]. However, some studies have highlighted the paradox (African or India enigma) between a high rate of *Helicobacter pylori* infection in the general population and a low incidence of gastric cancer [15, 16]. The delayed diagnosis highlighted in our work can be explained by the socio-cultural interpretation of the disease in Africa, which is most often attributed to witchcraft or bad luck. The patient's first reflex is then to visit a traditional healer who will ward off this bad luck through ritual sacrifices and indigenous treatments. The forms received in “Western” hospital consultations are therefore most often advanced [17]. Thus in our study, 67.5% of our patients had weight loss, 80% had a WHO performance index of at least 3, and 84.1% were at the TNM III or IV stage. Also, 25% of our patients had a Troisier´s sign and 35.8% had a palpable epigastric mass. These results corroborate with those of other African studies, where 60% of patients had an epigastric mass at the time of diagnosis [6], 78.58% having metastases[7], and 36% at TNM IV stage [8].

The GC in our study was most often located on the antrum (44.2%) and was adenocarcinoma in 87.5%. This result is similar to those reported by other African studies [8, 18, 19]. Only 86% of patients could perform an abdominal CT-scan. This is due to the absence of a national health insurance system, as patients have to finance their medical care. No gastric endoscopic ultrasound could be done because no health center in the city of Yaoundé and even in Cameroon as a whole is equipped for this examination to date. Delay in consultation linked to cultural/financial considerations, poverty with lack of health coverage, and insufficient technical platform of hospitals, such a triad of difficulties plumb the prognosis of GC in Africa. Our study shows that the prognosis of GC in our context is bad with a 3 and 5-year survival rate of 10.1 and 4.6%respectively. Our study identified 4 factors significantly and independently linked to a risk of mortality. Three of these factors (WHO Stage 3 performance index, palpable epigastric mass on clinical examination, presence of liver metastases) are linked to the diagnostic delay already mentioned. Several studies have already highlighted the poor prognosis of locally advanced or metastatic forms of gastric cancer [20-24]. With an early diagnosis, the prognosis is better, with a 5-year survival of up to 90% [25]. Our work highlights this fact because the early detected forms (TNM stages I and II) had a better prognosis than the others (TNM stages III and IV). Similarly, patients in whom curative treatment could have been implemented (therefore detected in the non-metastatic stages) had a better prognosis. Early detection with systematic screening from the age of 40 and awareness campaigns encouraging patients to consult quickly are lines of thought to improve the prognosis of GC in our country.

The fourth risk factor for mortality identified in our study was the pyloric location of the tumor. Indeed, in this location, the tumor will be rapidly occlusive with an alteration in the general condition which has already been identified as a risk factor for mortality. To the best of our knowledge, our study is the first to assess survival as well as mortality risk factors ofGC in our country. Mortality risk factors identified in this work seems to be common in many others black African countries, which has a fairly similar socio-economic and health situation as in our own. However, prospective cohort studies may better analyze the factors highlighted in this work. This study can also be a tool in the hands of decision-makers, to improve the prognosis of this cancer in our environment by setting up a national cancer registry and improving hospitals technical platform.

## Conclusion

Clinical epidemiology of GC in our study is comparable to those of other African studies with a predominance of locally advanced/metastatic forms. Prognosis is grim with diagnostic delay behind all of the identified mortality risk factors. Creation of a national cancer registry, improvement of hospitals' technical platform, and setting up of awareness campaigns are draft solutions to improve the survival of GC in our environment.

### What is known about this topic

- The estimated incidence rate of gastrci cancer in Africa is low;
- At the time of diagnosis, metastatic forms are predominant;
- The prognosis of gastric cancer in Africa is poor.

### What this study adds

- The most common risk factor for gastric cancer in Cameroon is Helicobacter pylori infection;
- Survival rate of gastric cancer in cameroon at 3 and 5 years is 10.1% and 4.6%, respectively;
- Variables independently associated with overall survival included: WHO stage 3 performance status, palpable epigastric mass on examination, pyloric localization and liver metastasis.
